# Stimulus-Induced Histone H3 Citrullination and Neutrophil Extracellular Trap Formation Are Impaired in Patients with Proliferative Diabetic Retinopathy

**DOI:** 10.3390/biomedicines14102148

**Published:** 2026-09-23

**Authors:** Beatriz Buentello-Volante, Fátima Sofía Magaña-Guerrero, Norma Angélica Magaña-Guerrero, José Luis Rodriguez-Loaiza, Oscar Vivanco-Rojas, Alfredo Domínguez-López, Yonathan Garfias

**Affiliations:** 1 Cell and Tissue Department, Research Unit, Institute of Ophthalmology Conde de Valenciana, Mexico City 06800, Mexico; bbuentello@institutodeoftalmologia.org (B.B.-V.); fatima.magana@institutodeoftalmologia.org (F.S.M.-G.); namg6398@gmail.com (N.A.M.-G.); oscarv@bq.unam.mx (O.V.-R.); adominguezl@facmed.unam.mx (A.D.-L.); 2 Department of Biochemistry, Faculty of Medicine, Universidad Nacional Autónoma de México, Av. Universidad 3000, Mexico City 04510, Mexico; 3 Department of Retina, Institute of Ophthalmology Conde de Valenciana, Mexico City 06800, Mexico; jose.rodriguez@institutodeoftalmologia.org

**Keywords:** proliferative diabetic retinopathy, neutrophil extracellular traps (NETs), histone H3 citrullination, PADI4, immune dysfunction

## Abstract

**Background**: Proliferative diabetic retinopathy (PDR) is the most advanced stage of diabetic retinopathy (DR). Although spontaneous release of Neutrophil Extracellular Traps (NETs) has been reported in DR patients, the capacity of neutrophils from PDR patients to undergo inducible NET formation remains unexplored. This study aimed to evaluate inducible NET release and the underlying regulatory mechanisms in PDR. **Methods**: We performed a case–control study including 9 nondiabetic controls, 17 patients with type 2 diabetes (T2D) without DR, and 15 patients with PDR. Peripheral neutrophils were isolated and stimulated with ionomycin to assess NET release and histone H3 citrullination (H3Cit). Peptidylarginine deiminase 4 (*PADI4*)promoter methylation and mRNA expression were analyzed, together with the surface expression of the activation markers CD11b and CD66b. An ex vivo high-glucose model was used to examine the effect of persistent hyperglycemia on NET induction. **Results**: Compared with those from controls and T2D patients, neutrophils from PDR patients exhibited significantly reduced inducible NET release and impaired H3Cit. The NET index (stimulated/nonstimulated ratio) was markedly lower in the PDR group. Despite increased *PADI4* mRNA expression in PDR neutrophils, promoter methylation, as well as that of cell activation markers, remained unchanged. Notably, persistent hyperglycemia in healthy neutrophils reproduced the impaired NET release and reduced H3Cit observed in PDR. **Conclusions**: Neutrophils from PDR patients display selective defects in inducible NET release and histone citrullination, which are likely driven by chronic hyperglycemia and metabolic stress rather than epigenetic silencing of *PADI4*. These findings are consistent with impaired inducible neutrophil responsiveness in PDR and suggest that altered regulation of PAD4-dependent histone citrullination may contribute to this phenotype, warranting further mechanistic investigation.

## 1. Introduction

Diabetic retinopathy (DR), a microvascular complication of type 2 diabetes (T2D), represents the principal global cause of preventable blindness. Its most advanced manifestation, proliferative diabetic retinopathy (PDR), is pathologically characterized by retinal neovascularization, subsequent fibrosis, and extensive ischemia. These pathological changes collectively contribute to significant visual impairment and, if left untreated, result in irreversible blindness [1]. While the pathophysiology of DR has classically focused on hyperglycemia-induced vascular damage, emerging evidence highlights a significant role for immune dysregulation and chronic inflammation in disease progression [1], particularly involving innate immune cells such as neutrophils [2,3]. Neutrophils contribute to host defense through multiple mechanisms, including degranulation, phagocytosis, and the formation of neutrophil extracellular traps (NETs), among many others. NETs are composed of a web-like meshwork of chromatin and antimicrobial proteins expelled during a specialized form of cell death known as NETosis [4]. While NETs serve as potent antimicrobial structures, their aberrant release has been linked to microvascular injury [5], endothelial dysfunction [6], and sterile inflammation [7], all of which are relevant to diabetic complications. Recent studies have shown elevated NET markers, including citrullinated histone H3 (H3Cit) and neutrophil elastase (NE), in the circulation and retinal tissues of patients with PDR [8,9]. Peptidylarginine deiminase 4 (PAD4), encoded by the *PADI4* gene, is a critical enzyme that participates in the formation of NETs. PAD4 catalyzes the citrullination of histone H3, a modification critical for chromatin decondensation and subsequent NET release [10]. The regulation of PAD4 is calcium-dependent and tightly linked to the metabolic and inflammatory state of cells. Hyperglycemia and oxidative stress have been shown to modulate PAD4 activity, either by impairing calcium homeostasis [11] or altering intracellular signaling cascades [12]. However, whether PAD4 activity is impaired in neutrophils from individuals with PDR and how this activity contributes to defective NET formation remain poorly understood. Moreover, the transcriptional and epigenetic regulation of *PADI4* under diabetic conditions is an area of active investigation. DNA methylation of the *PADI4* promoter has been implicated in autoimmune diseases [13], but its role in diabetes and DR is unknown. Exploring whether PAD4 dysregulation stems from transcriptional repression, epigenetic silencing, or metabolic suppression is essential for understanding the functional capacity of neutrophils in diabetes. Despite reports of increased spontaneous NET release in diabetes and its complications [14], little is known about inducible NET formation capacity in PDR neutrophils, a defining characteristic of robust immune responsiveness. It remains unclear whether these cells are primed for proinflammatory activity or display signs of exhaustion and unresponsiveness because of prolonged stimulation, a state recently described as innate immune tolerance [15]. This is further complicated by the observation that surface activation markers such as CD11b and CD66b, which are involved in adhesion and degranulation, may not correlate with functional NET release, which can be either diminished or exaggerated independently of their expression [16]. This study investigated the regulation of NET release in peripheral isolated neutrophils upon NET activation by comparing the responses among nondiabetic controls, subjects with T2D without DR, and patients with PDR. We evaluated multiple parameters, including NET release and histone H3Cit levels following ionomycin-induced activation, *PADI4* promoter methylation and gene expression, the surface expression of the activation markers CD11b and CD66b, and the effect of persistent hyperglycemic exposure on NET release, using an ex vivo model.

## 2. Materials and Methods

This was a case–control study conducted at the Institute of Ophthalmology “Conde de Valenciana” Foundation. This cohort is derived from a previously reported cohort, with slight modifications [15]. The study adhered to the principles of the Helsinki Declaration and received approval from the “Conde de Valenciana” Institute of Ophthalmology IRB (CEI-2020/01/01). All participants provided written informed consent. The control subjects were healthy volunteers with no history of T2D or systemic diseases, including autoimmune or renal diseases, hypertension, or active infections. The definition of patients with T2D adhered to the current criteria established by the American Diabetes Association (ADA). Eligible participants included individuals of both sexes aged greater than 18 years. The exclusion criteria included patients who presented with inflammatory-related diseases (excluding diabetes), such as active or chronic infections, as well as immunocompromised subjects. Furthermore, patients for whom retinal fundoscopy could not be performed because of conditions such as media opacity or cataracts were also excluded. Participants whose biological samples proved unsuitable for processing were excluded from the study. Mydriatic fundus photographs of both eyes were obtained for all patients using the Optos P200DTx imaging system (Optos plc, Dunfermline, Scotland, UK). A retina specialist, masked to patient information, independently classified all participants according to the Diabetic Retinopathy Disease Severity Scale and the International Clinical Diabetic Retinopathy Disease Severity Scale. The T2D participants were divided into two groups: those without DR (with solely T2D) and those with proliferative diabetic retinopathy (PDR). A 30 mL peripheral blood sample was obtained from each participant. Urinalysis and a comprehensive hematological analysis were performed on all participants. The estimated glomerular filtration rate (eGFR) was calculated using the MDRD-6 formula. Serum osmolality was calculated using the standard equation: Osm = 2(Na + K) + (Glucose/18) + (BUN/2.8). The fasting triglyceride-to-fasting glucose index (TyG) was calculated as follows: TyG index = ln [(fasting triglyceride concentration in mg/dL × fasting glucose concentration in mg/dL)/2]. To assess the effect of hyperglycemia, neutrophils from two independent healthy donors, who were not among the subjects initially recruited for the study, were incubated under normoglycemic or high-glucose conditions, followed by stimulation or left unstimulated. Each experimental condition was assessed in triplicate.

### 2.1. Peripheral Blood Neutrophil Isolation and NET Induction and Evaluation

Neutrophil isolation. Neutrophils were isolated from peripheral blood via density gradient centrifugation. The cell viability, which was assessed immediately after isolation using the trypan blue exclusion method, was 96%. In other assays, cells from all study groups were subsequently maintained under the same standardized 24-h incubation conditions, although endpoint viability was not included as a separate measurement. The procedure started with the collection of 20 mL of complete peripheral blood into heparinized tubes from individuals who had fasted for 6–8 h. This blood was carefully overlaid onto an equivalent volume of Polymorphoprep (Axis-Shield, Oslo, Norway) solution and subjected to centrifugation at 500× *g* for 35 min at room temperature (RT). The band containing neutrophils was then harvested and washed with 0.8 mL of ice-cold 0.1 M PBS. The remaining erythrocytes were lysed using a solution composed of 152.7 mM NH4Cl, 0.1 mM Na2EDTA, and 9.0 mM NaHCO3 (pH 7.2–7.4), followed by subsequent washes with 0.1 M PBS as needed. The purified neutrophils were finally suspended in Hank’s balanced salt solution (HBSS without calcium; Gibco, Thermo Fisher Scientific, Waltham, MA, USA) and maintained on ice until further experimentation. Neutrophil viability was determined using the trypan blue exclusion assay, and the purity of the cell preparation was confirmed by flow cytometric analysis, which revealed that ≥98% of the cells expressed both CD11b and CD15, and the viability was 96%. In vitro NET induction. For each subject, neutrophils were seeded onto poly-L-lysine (Sigma-Aldrich, St. Louis, MO, USA) precoated glass coverslips in 24-well plates at a density of 4 × 10^5^ cells per well. Adherence was allowed to increase at 37 °C under a 5% CO_2_ atmosphere for 20 min. After this, the cells were stimulated with ionomycin from Streptomyces conglobatus (1.25 µM) and calcium chloride (1.5 mM) (Sigma-Aldrich, St. Louis, MO, USA) for 1 h at 37 °C in a 5% CO_2_ atmosphere (S, stimulated condition), or the cells were incubated for only 1 h at 37 °C in a 5% CO_2_ atmosphere (NS, nonstimulated condition). Neutrophil extracellular traps (NETs) were fixed using a 4% p-formaldehyde (Sigma-Aldrich, St. Louis, MO, USA) solution for 10 min at RT and then were washed twice with 0.1 M PBS. For in vitro NET visualization, the samples were subjected to overnight incubation at 4 °C with continuous agitation in the presence of a rabbit anti-human neutrophil elastase (NE; Abcam, Cambridge, UK) primary antibody (1:100). As a negative control, primary antibodies were excluded. The samples were subsequently washed three times with PBS containing 0.05% Tween 20 (PBS-T) and then incubated for 2 h at RT with an Alexa Fluor 488-conjugated goat anti-rabbit IgG secondary antibody (1:800; Invitrogen-Thermo Fisher Scientific, Carlsbad, CA, USA). Following two washes with PBS-T, DNA was visualized by incubation with 25 μg/mL propidium iodide (PI) (Sigma--Aldrich, St. Louis, MO, USA). Finally, the specimens were mounted, and image acquisition was performed using an ApoTome II microscope (Carl Zeiss Microscopy GmbH, Jena, Germany) controlled by ZEN 3.4 (blue edition) software (Carl Zeiss, Jena, Germany). Induction of NETs under hyperglycemic conditions. Neutrophils were isolated from healthy donors and seeded onto poly-L-lysine-coated glass coverslips placed in 24-well plates at a density of 2 × 10^5^ cells per well. The cells were allowed to adhere for 20 min at 37 °C in a humidified atmosphere containing 5% CO_2_. Following adhesion, the cells were incubated for 24 h at 37 °C with 5% CO_2_ in RPMI medium (Invitrogen, Thermo Fisher Scientific, Carlsbad, CA, USA) supplemented with 2% fetal bovine serum (FBS) and either 5.5 mM or 25 mM glucose. After incubation, the neutrophils were either stimulated with ionomycin and calcium chloride for 1 h (Glc [+] + iono) or left unstimulated (only Glc [+]). NETs were fixed with 4% p-formaldehyde for 10 min at RT, followed by two washes with PBS. To visualize NETs by immunofluorescence, the samples were incubated overnight at 4 °C with gentle agitation in the presence of an anti-histone H3 (H3Cit) (citrulline R2 + R8 + R17) antibody (Abcam, Cambridge, UK; 1:300). Negative controls were prepared by omitting the primary antibody. After three washes with PBS containing 0.05% Tween 20 (PBS-T), the samples were incubated for 2 h at RT with an Alexa Fluor 488-conjugated goat anti-rabbit IgG secondary antibody (1:800). Finally, the DNA was counterstained using PI at 25 μg/mL, followed by two additional washes with PBS-T. The samples were mounted, and image acquisition was performed using an ApoTome II microscope controlled by ZEN 3.4 software for NET analysis. In vitro NET quantification. NET area release was quantified by analyzing three randomly selected images from each sample across three independent experiments, which were captured using an ApoTome II microscope and Carl Zeiss ZEN 3.4 (blue edition) software. A machine learning algorithm, adapted with minor modifications from a previously published protocol [14], was implemented for the detection and subsequent quantification of NET structures. To maintain analytical homogeneity, only image fields containing 80 nuclei, identified by PI staining, were included in the analysis. The Intellesis Trainable Segmentation module within Carl Zeiss ZEN 3.4 (blue edition) was utilized for total NET area quantification (area/µm^2^), with nuclei and background regions excluded. To accurately assess intraindividual activation while minimizing intersubject variability, a NET index was calculated for each participant. Absolute NET areas were quantified separately under nonstimulated and stimulated conditions for each participant. In addition, the NET index was calculated as the stimulated-to-nonstimulated ratio (S/NS) to summarize the relative inducible response in relation to each participant’s basal NET formation. Because this ratio is influenced by both the stimulated response and the basal value, it was interpreted together with the corresponding absolute measurements rather than as an independent measure of NET-forming capacity. The resulting indices were reported as the mean ± standard error of the mean (SEM).

### 2.2. Western Blot Assays

To assess the expression of H3Cit, human neutrophils (1.9 × 10^6^ cells) subjected to the indicated conditions were lysed and analyzed by Western blot. Protein lysates were resolved on 4–20% SDS-PAGE gels (Bio-Rad Laboratories, Hercules, CA, USA) and transferred to nitrocellulose membranes (Bio-Rad Laboratories, Hercules, CA, USA). The membranes were blocked for 1 h at RT using 5% BSA or low-fat milk in TBS-T (1× TBS with 0.1% Tween-20), as required for the primary antibody. Following blocking, the membranes were incubated overnight at 4 °C with H3Cit (citrulline R2 + R8 + R17) antibody (Abcam, Cambridge, UK). After being washed with TBS-T, the membranes were incubated with an HRP-conjugated goat anti-rabbit secondary antibody (Jackson ImmunoResearch, West Grove, PA, USA) for 2 h at RT. Signal detection was performed using chemiluminescent substrates (SuperSignal West Pico or West Femto, Thermo Scientific, Rockford, IL, USA). The signal was detected via G-BOX equipment and Gene Snap software version 7.12.06 (Sygene, Cambridge, UK). Anti-Histone H3 (Abcam, Cambridge, UK) antibody was used as a loading control.

### 2.3. PADI4 Promoter Methylation Assays

Genomic DNA (gDNA) was extracted from 10^6^ human peripheral neutrophils using the AllPrep DNA/RNA Micro Kit (Qiagen, Hilden, Germany). gDNA (100 ng) was bisulfite-converted with the EpiTect Fast 96 Bisulfite Conversion Kit (Qiagen) according to the manufacturer’s instructions. Bisulfite treatment converts unmethylated cytosines to uracils while leaving methylated cytosines unchanged, enabling discrimination of methylation status. Methylation of the PADI4 promoter was analyzed by quantitative methylation-specific PCR (qMSP) with high-resolution melting (HRM). The primer sequences and cycling parameters are provided in the Table A1, Appendix A. PCRs (25 µL) contained KAPA PROBE FAST qPCR Master Mix (2×) Universal (Kapa Biosystems, Wilmington, MA, USA), 0.5 µM primers, and 2 µL bisulfite-modified DNA (~10 ng). Standard curves (0–100% methylation) were generated from mixtures of fully methylated and unmethylated DNA (EpiTect PCR Control DNA Set; Qiagen). Methylation levels of unknowns were estimated by comparing melting peak Tm values to standards. Negative (no-template) and bisulfite conversion controls were included in all the runs. Reactions were performed in triplicate.

### 2.4. RT-qPCR Assays

Total RNA was isolated from peripheral blood neutrophils using an AllPrep DNA/RNA Kit (Qiagen, Hilden, Germany) according to the manufacturer’s instructions. RNA concentration and purity were assessed with a NanoDrop 2000 spectrophotometer (Thermo Fisher Scientific, Waltham, MA, USA). Ten nanograms of RNA were used per reaction, and mRNA expression was quantified by one-step real-time RT-PCR using the KAPA SYBR^®^ FAST One-Step qRT-PCR Kit (Kapa Biosystems, Roche Molecular Systems, Inc., Brackenfell, South Africa). The primer sequences and RT-qPCR cycling parameters are provided in the Table A2, Appendix A. Reactions were performed in triplicate. PADI4 expression was measured by real-time RT-qPCR and normalized to that of β2-microglobulin (B2M). For each sample, ΔCt was calculated as CtPADI4 − CtB2M. For graphical presentation, normalized expression is expressed as −ΔCt, which is mathematically equivalent to log_2_(2^−ΔCt^), such that higher values indicate greater PADI4 expression relative to B2M. No external sample or control-group calibrator was used.

### 2.5. Flow Cytometry Assays

Peripheral blood was collected into sodium citrate-anticoagulant tubes and processed within 2 h of collection. For each subject, 100 µL of whole blood was incubated under nonstimulated (NS) or stimulated (S) conditions. Following incubation, the cells were surface stained with the following fluorochrome-conjugated monoclonal antibodies: PE-conjugated anti-CD11b–PE (BD Biosciences, San Jose, CA, USA) and BV450-conjugated anti-CD66b (BD Biosciences, San Jose, CA, USA) for 30 min at 4 °C in the dark. Red blood cells were lysed using 1X BD FACS Lysing Solution (BD Biosciences, San Jose, CA, USA) according to the manufacturer’s protocol. The samples were suspended in 100 µL of PBS. Isotype-matched control antibodies were used to assess nonspecific binding. Data acquisition was carried out on a FACS Lyric flow cytometer (BD Biosciences, San Jose, CA, USA), and 1 × 10^5^ events of the polymorphonuclear region were collected per sample. Neutrophils were gated on the basis of forward scatter (FSC) and side scatter (SSC) characteristics, and marker expression was analyzed using FlowJo software (v10.8; BD Life Sciences). The results are expressed as the mean fluorescence intensity (MFI) for each marker under NS and S conditions. Data are presented as the mean ± SEM, and statistical comparisons were performed between conditions and groups.

### 2.6. Statistical Analysis

Prior to analysis, data distribution was assessed using the Shapiro–Wilk and Kolmogorov–Smirnov tests, which indicated departures from normality. Accordingly, between-group comparisons were performed using the nonparametric Kruskal–Wallis test, with statistical significance set at *p* < 0.05. Associations between the NET index and clinical and biochemical variables were evaluated using one-tailed Spearman rank correlation tests. The expected direction of these associations was defined a priori on the basis of previous clinical evidence showing that spontaneous NET release increases with diabetes duration, fasting glucose, HbA1c, and plasma osmolality and decreases with eGFR [14], as well as experimental evidence indicating that high-glucose exposure induces a constitutively activated neutrophil state with reduced responsiveness to subsequent stimulation [17]. We therefore hypothesized that worsening metabolic and renal status would be associated with a lower inducible NET response, whereas indicators of preserved metabolic and renal function would be associated with a higher NET index. These correlations were considered secondary, hypothesis-driven analyses. All the statistical analyses and graphical representations were performed using GraphPad Prism version 8 (GraphPad Software, La Jolla, CA, USA).

## 3. Results

A total of 41 subjects were included: 9 controls, 17 with type 2 diabetes (T2D) without diabetic retinopathy, and 15 with proliferative diabetic retinopathy (PDR). As shown in Table 1, the groups differed significantly in terms of demographic, clinical, and metabolic parameters, including age; diabetes duration; systolic blood pressure; fasting glucose level; HbA1c level; renal function markers (urea, creatinine, eGFR, BUN); lipid profile (HDL-C, triglyceride, atherogenic index); and TyG, uric acid, bilirubin, and serum osmolality levels. Notably, diabetes duration and renal function parameters also differed between the T2D and PDR groups.

NET release patterns differed across groups (Figure 1A,B). Analysis of absolute NET areas revealed distinct stimulation–response profiles (Figure 1B). Ionomycin markedly increased NET formation in control neutrophils, whereas neutrophils from individuals with T2D exhibited only a modest but nonsignificant increase in NET formation. In contrast, after stimulation, the number of neutrophils in PDR was elevated, resulting in increased spontaneous NET formation under basal conditions, followed by a paradoxical reduction rather than a further increase. Consequently, the significantly lower NET index observed in the PDR group than in the control group (Figure 1C) reflects both elevated basal NET formation and the lack of an additional inducible response, rather than merely an artifact of the ratio calculation.

Histone H3 citrullination increased upon stimulation in controls and T2D patients but not in PDR patients, in which basal H3Cit levels were elevated but unresponsive to stimulation (Figure 2A,B). The NET index of H3Cit was significantly lower in PDR neutrophils than in control neutrophils (Figure 2C).

The NET index correlated negatively with age, diabetes duration, systolic blood pressure, fasting glucose, HbA1c, renal dysfunction (urea, creatinine, BUN), TyG, uric acid, and serum osmolality, while positive correlations were found between the eGFR and HDL-C (Table 2). These associations link impaired inducible NET release with poor glycemic control and renal impairment.

Epigenetic and transcriptional analyses (Figure 3) revealed no significant group differences in *PADI4* promoter methylation (Figure 3A), but *PADI4* mRNA (Figure 3B) was significantly upregulated in PDR patients compared with controls. In contrast, the surface activation markers CD11b and CD66b increased upon stimulation in all groups, but the differences were not significant, indicating that the neutrophil activation capacity was preserved (Figure 3C,D). Representative flow cytometry histograms illustrating the fluorescence-intensity distributions of CD66b and CD11b under non-stimulated and stimulated conditions in the Control, T2D, and PDR groups are provided in Supplementary Figure S1.

Prolonged exposure to high glucose resulted in a coordinated reduction in ionomycin-induced NET formation and H3Cit responses. After 24 h of culture in medium containing 25 mM glucose, compared with control cells, neutrophils formed fewer NETs (Figure 4A). Ionomycin-associated H3Cit was also reduced, as demonstrated by both immunofluorescence (Figure 4A) and Western blot analysis (Figure 4B). Thus, prolonged high-glucose exposure consistently affects both extracellular chromatin release and histone H3 citrullination, a modification associated with PAD4-mediated NET formation.

Acute exposure to 25 mM glucose for 1 h also reduced ionomycin-induced NET formation and H3Cit fluorescence intensity (Figure 5A). However, Western blot analysis did not detect a corresponding reduction in the H3Cit/H3 ratio (Figure 5B). Therefore, the effect of acute high-glucose exposure on H3Cit was assay-dependent, whereas after 24 h, the reduction was consistently detected by both immunofluorescence and Western blot analysis. Collectively, these findings suggest that prolonged high-glucose exposure results in broader and more consistently detectable impairment of the NET-associated response.

## 4. Discussion

This study reveals a complex pattern of neutrophil dysregulation in patients with PDR characterized by impaired inducible NET formation, altered citrullination dynamics, and preserved surface activation capacity. Our integrated approach—encompassing immunofluorescence microscopy, Western blotting, epigenetic profiling, gene expression, and flow cytometry—provides robust evidence that chronic hyperglycemia disrupts PAD4-dependent neutrophil responses through mechanisms that are independent of *PADI4* promoter methylation but potentially driven by metabolic modulation of enzyme activity. We report that NET release is significantly attenuated upon stimulation of neutrophils derived from PDR patients. Unlike neutrophils obtained from control subjects, which showed robust NET release following ionomycin/calcium activation, neutrophils from PDR patients exhibited a paradoxical decrease in NET production upon stimulation, despite the presence of elevated baseline NETs. This blunted response, quantified through a reduced NET index (S/NS ratio), reveals a previously unrecognized defect in inducible NETs during advanced diabetic complications. The aberrant increase in spontaneous NET release at rest observed in different stages of DR [14], including PDR, together with the reduced capacity for further activation seen in diabetes [18], suggests a preactivated neutrophil phenotype [19]. In this context, because the NET index is calculated as the stimulated-to-nonstimulated ratio, the elevated basal NET formation observed in PDR contributes mathematically to its lower value. The index should therefore be interpreted as a measure of relative inducible response rather than as an isolated measure of NET-forming capacity. Nevertheless, examination of the absolute measurements revealed that compared with their basal level, PDR neutrophils failed to increase NET formation following stimulation but instead exhibited a reduction in NET formation. Together with the concordant reduction in stimulus-induced H3 citrullination, these findings indicate that the lower NET index is not explained solely by an elevated denominator but reflects impaired responsiveness to further stimulation.

This reflects a state of functional exhaustion in proliferative PDR [20], where neutrophils remain chronically activated but fail to mount additional responses [21]. Such phenotypes have been documented in other chronic inflammatory conditions, including sepsis and systemic autoimmune diseases [15], and may reflect persistent exposure to low-grade systemic inflammation or circulating damage-associated molecular patterns (DAMPs) in advanced diabetes [22]. However, the cross-sectional design of our study does not allow us to determine whether the reduced inducible NET formation observed in PDR is a causal factor contributing to disease progression or a consequence of advanced retinal pathology. Both possibilities remain biologically plausible: chronic hyperglycemia and sustained inflammation may lead to functional exhaustion of neutrophils, while impaired NET production could also contribute to a dysregulated inflammatory environment. Longitudinal studies will be needed to clarify the directionality of this association. In addition, previous studies have reported that NET levels are significantly increased in the serum of diabetic patients, especially in proliferative diabetic retinopathy (PDR) patients, and that high glucose levels dramatically increase NET production in diabetic individuals over time [3]. We previously reported that NETs participate in the inflammatory mechanisms of neutrophils and are correlated with clinical variables such as HbA1c levels, the eGFR, and the duration of diabetes, which are themselves associated with the development and severity of diabetic retinopathy [15]. In the present study, considerable heterogeneity across groups—particularly in terms of age, diabetes duration, glycemic control, and renal function—should therefore be acknowledged as a potential limitation. Because these factors are also associated with the NET index, their contribution to, or modification of, the impaired inducible NET response observed in PDR cannot be entirely excluded. Despite limiting the attribution of this altered response specifically to PDR, these observations support the idea that NET dysregulation reflects underlying metabolic and inflammatory deterioration in diabetes, although NETs are not specific markers of diabetic retinopathy.

Citrullination of histone H3, a posttranslational modification catalyzed by PAD4, is a key chromatin-remodeling event essential for NET release and has been directly implicated in the pathogenesis of diabetes [11]. In the present study, neutrophils from control subjects showed the expected pattern of increased H3Cit following ionomycin/calcium stimulation. In contrast, the observed reduction in H3Cit following stimulation may be consistent with altered regulation of PAD4-dependent histone citrullination at the posttranscriptional or functional level. However, because PAD4 protein expression and enzymatic activity were not directly assessed, this interpretation remains tentative, and alternative mechanisms cannot be excluded. This dissociation strongly suggests that the citrullination machinery in PDR neutrophils is dysregulated, with impaired responsiveness to calcium-dependent activation. These findings are consistent with the notion that chronic hyperglycemia may lead to calcium channel dysregulation [23,24], which could impair PAD4 enzyme function. Interestingly, neutrophils from individuals with T2D displayed only mild alterations in both NET formation and H3Cit expression, suggesting that this dysregulation worsens with disease progression and is particularly prominent in the PDR stage. These data point toward a staged immune dysfunction in DR, where immune imbalance becomes more severe in tandem with microvascular damage [25]. One of the outstanding findings of this study is the relationship between *PADI4* gene promoter methylation and gene expression. In contrast to previous studies reporting an inverse correlation between *PADI4* promoter methylation and anti-PAD4/ACPA antibody levels, as well as disease activity scores in rheumatoid arthritis [13], our findings show that although *PADI4* promoter methylation levels did not differ significantly among the control, T2D, and PDR groups, *PADI4* mRNA expression was significantly elevated in neutrophils from PDR patients, suggesting that epigenetic silencing via DNA methylation is not the mechanism that regulates *PADI4* expression, at least in this context. The observed upregulation may instead be driven by inflammatory transcription factors [26,27] or hypoxia-inducible elements [28], which are hallmarks of the diabetes microenvironment. However, despite this increase in transcription, PAD4-dependent histone citrullination remains defective in neutrophils from PDR patients, suggesting possible posttranscriptional or functional dysregulation of the PAD4 pathway, possibly through the activation of PKCα instead of PKCζ [29]. These findings highlight an important concept: gene expression levels may not necessarily predict enzymatic or cellular function, particularly under conditions of chronic stress such as PDR. These findings underscore the need for functional validation of transcriptomic data in immune studies of diabetes and its complications. To determine whether the observed defects in NET release and citrullination reflected global impairment of neutrophil activation, we assessed the expression of the classical surface activation markers CD11b and CD66b. These molecules reflect integrin activation and degranulation, respectively. In all groups, including the PDR group, the expression of these markers significantly increased upon stimulation, indicating that early neutrophil activation steps remain functionally intact. These findings suggest that the defect is specific to nuclear events downstream of surface activation, further supporting the hypothesis of selective PAD4 pathway dysfunction in diabetes [30]. The preservation of these pathways also indicates that neutrophils from PDR patients retain the ability to adhere and migrate, which may contribute to tissue infiltration and damage [31] despite impaired NET release.

To experimentally model the influence of glycemic status, we exposed neutrophils obtained from healthy individuals to hyperglycemic conditions (25 mM glucose) for 24 h (persistent) or 1 h (acute). High-glucose experiments revealed exposure time-dependent differences in the NET-associated response. After 1 h, high glucose reduced ionomycin-induced NET formation and H3Cit fluorescence, although a corresponding reduction in the H3Cit/H3 ratio was not detected by Western blot. In contrast, after 24 h, reduced NET formation was accompanied by decreased H3Cit levels, as measured by both immunofluorescence and Western blotting. Thus, prolonged high-glucose exposure resulted in more consistent impairment of histone citrullination across both assays. PAD4-mediated histone citrullination contributes to chromatin decondensation, but NET release involves additional coordinated nuclear and cellular events [32,33,34]. Therefore, the discrepancy between H3Cit measurements after acute exposure precludes attributing the reduced NET response specifically to PAD4 inhibition or to a particular downstream mechanism. Further time-course studies incorporating direct measurements of PAD4 activity and intermediate stages of NET formation are needed to elucidate the mechanisms underlying these time-dependent effects. These findings suggest that persistent hyperglycemia impairs PAD4-dependent NET formation, likely through oxidative stress, epigenetic modulation, or metabolic reprogramming [17]; however, the absence of osmotic control limits our ability to attribute this effect specifically to glucose. Although pharmacological modulation of PAD4 has been proposed [35], our findings do not support the notion that it enhances PAD4 activity or induces NET formation in PDR. Given the contribution of excessive basal NETosis to diabetic vascular injury [30], the blunted inducible response could represent an adaptive mechanism that limits further tissue damage. Whether this response is protective or contributes to impaired host defense and wound healing requires further investigation. Finally, our correlation analyses revealed significant inverse correlations between NET release and key clinical parameters, including age, duration of diabetes, blood pressure, HbA1c level, urea level, creatinine level, BUN level, and uric acid level, along with a positive correlation between the eGFR and HDL-C level. These associations suggest that NET dysfunction is strongly linked to poor glycemic control and declining renal function [36], both of which are known risk factors for PDR progression. These findings highlight the potential value of the NET index as a biomarker of immune competence and systemic metabolic balance in diabetes. It also supports the broader concept that chronic and systemic inflammation derangement leads to innate immune exhaustion [37], with significant implications for host defense and microvascular inflammation seen in T2D complications such as DR.

Our results indicate that although renal function parameters are significantly correlated with the NETs Index, the reduced inducible NET formation observed in PDR patients remains evident even when renal function status is considered. These findings suggest that the altered NET response is likely part of a broader systemic immune phenotype associated with PDR, although renal dysfunction may contribute as a modifying factor. Previously, we reported that compared with nondiabetic subjects, subjects with the most advanced stages of retinopathy, such as moderate and severe nonproliferative diabetic retinopathy as well as PDR, had significantly lower eGFRs [15].

This study provides novel insights into the immunometabolic dysfunction of neutrophils in the context of PDR. By integrating functional, transcriptional, and epigenetic analyses, we demonstrate that defective NET formation in PDR is not a consequence of classical activation failure or gene silencing but instead stems from a complex interplay between hyperglycemia and downstream PAD4-mediated signaling. These findings add evidence suggesting that innate immune tolerance [15] contributes to the progression of diabetic microvascular complications. From a clinical standpoint, our results suggest that the NET index and H3Cit level may serve as functional biomarkers [14] for immune competence and systemic metabolic stress in patients with diabetes. Furthermore, these findings raise the possibility that targeted modulation of PAD4 activity through metabolic control or pharmacologic intervention could restore neutrophil function and potentially mitigate secondary infections or exacerbated inflammatory responses in PDR [35]. Future longitudinal studies following diabetic patients over time will be essential to determine whether NET dysregulation precedes renal decline and PDR development or whether it emerges because of advanced metabolic and inflammatory deterioration. Understanding and reversing immune exhaustion in diabetes may therefore represent a paradigm shift in managing diabetic complications beyond glycemic control alone.

## 5. Conclusions

Collectively, our data reveal that PDR is associated with profound, selective defects in NET formation and histone citrullination, despite intact early neutrophil activation pathways. This dysregulation is not due to epigenetic silencing of *PADI4* but may be driven by persistent hyperglycemia and inflammatory stress that impair PAD4 function.

These findings contribute to our understanding of neutrophil dysfunction in diabetic retinopathy through the identification of an altered inducible NET response and possible dysregulation of PAD4-dependent histone citrullination in PDR. Future studies should determine whether this phenotype is reversible and whether it represents a protective adaptation that limits further retinal vascular injury or a dysfunctional state associated with impaired host defense and wound healing.

## Figures and Tables

**Figure 1:**
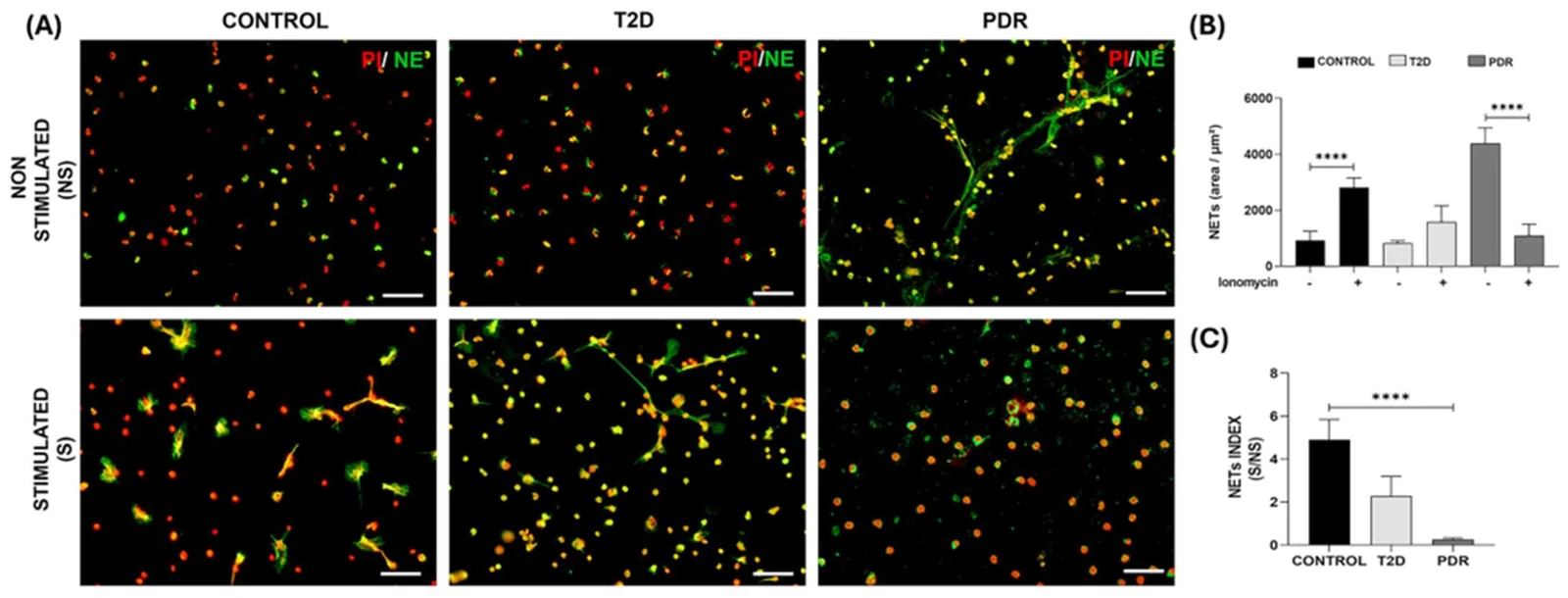
NET release was inhibited in neutrophils from PDR patients after stimulation. Peripheral neutrophils from control, T2D (T2D without diabetic retinopathy) and PDR (proliferative diabetic retinopathy) subjects were either stimulated with ionomycin or left unstimulated, and NET release was assessed. Representative micrographs of nonstimulated (NS) (upper panels) and stimulated (S) neutrophils (lower panels) in different groups (**A**). The total NET release areas in the nonstimulated (−) and stimulated (+) conditions of each group were compared (**B**). NET index comparisons between groups were performed (**C**). PI, propidium iodide; NE, neutrophil elastase. The bars represent the mean (±SEM). **** *p* < 0.0001. Scale bar = 20 μm.

**Figure 2:**
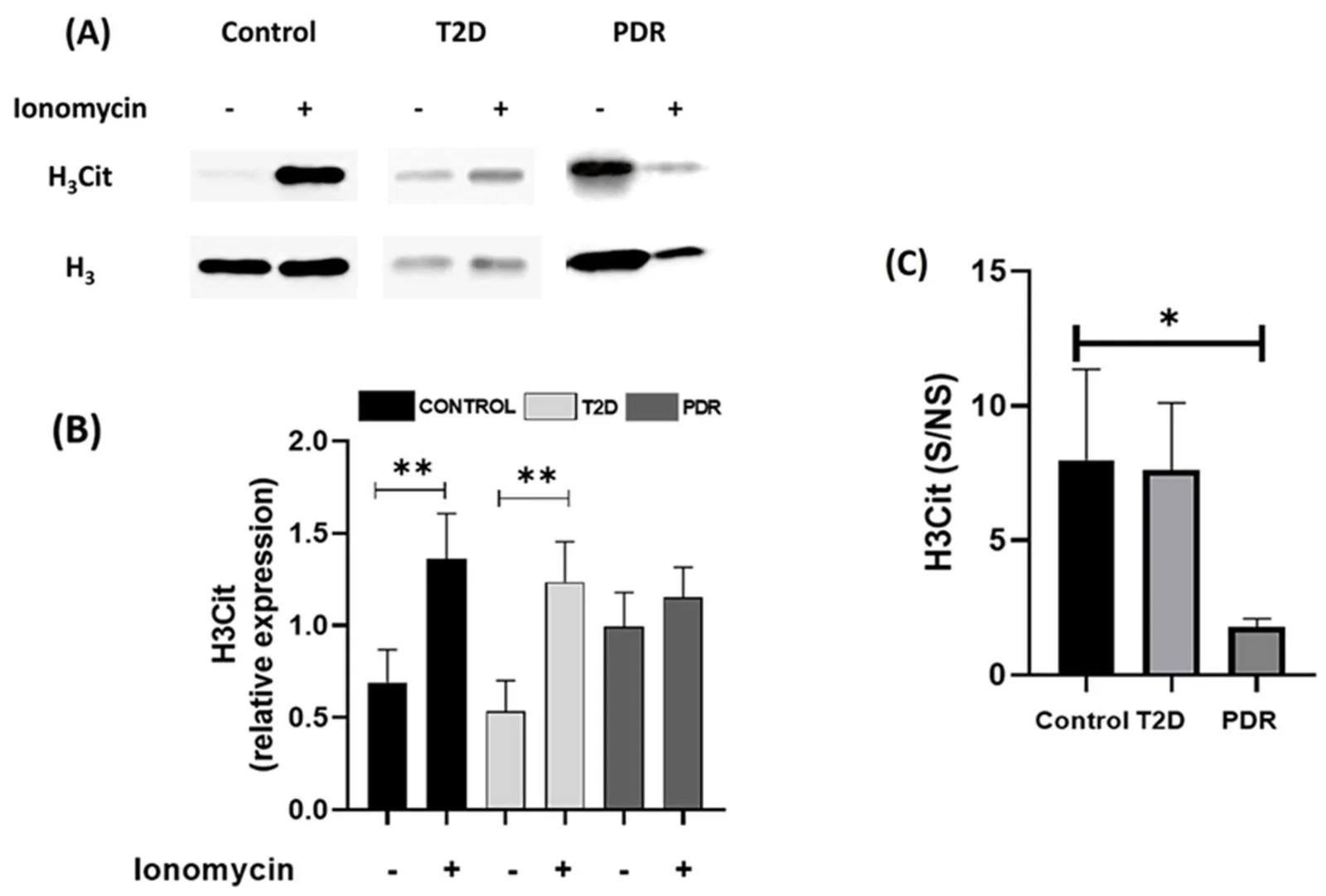
Stimulus-induced histone H3 citrullination is impaired in neutrophils from patients with proliferative diabetic retinopathy. Representative Western blots showing the expression of citrullinated histone H3 (H3Cit) and total histone H3 (H3) (loading control) in peripheral neutrophils from nonstimulated (NS) and stimulated (S, ionomycin-treated) control subjects, individuals with T2D (T2D, without diabetic retinopathy), and patients with proliferative diabetic retinopathy (PDR). Representative Western blot images of citrullinated histone H3 (H3Cit) and total histone H3 (H3, loading control) in neutrophils from control, T2D and PDR subjects under NS and S conditions. S notably upregulated the citrullination of H3 in control subjects and T2D patients, whereas it was not markedly modified in PDR subjects. Note that H3Cit expression was quantified by densitometry and normalized to the corresponding total H3 signal. Therefore, differences in absolute H3Cit band intensity should be interpreted relative to the total H3 loading control (**A**). Comparison of H3Cit relative expression among control, T2D and PDR subjects under S and NS conditions (**B**). Comparison of the S/NS ratios among control, T2D and PDR subjects (**C**). The bars represent the mean (±SEM). * *p*  <  0.05, ** *p* < 0.001.

**Figure 3:**
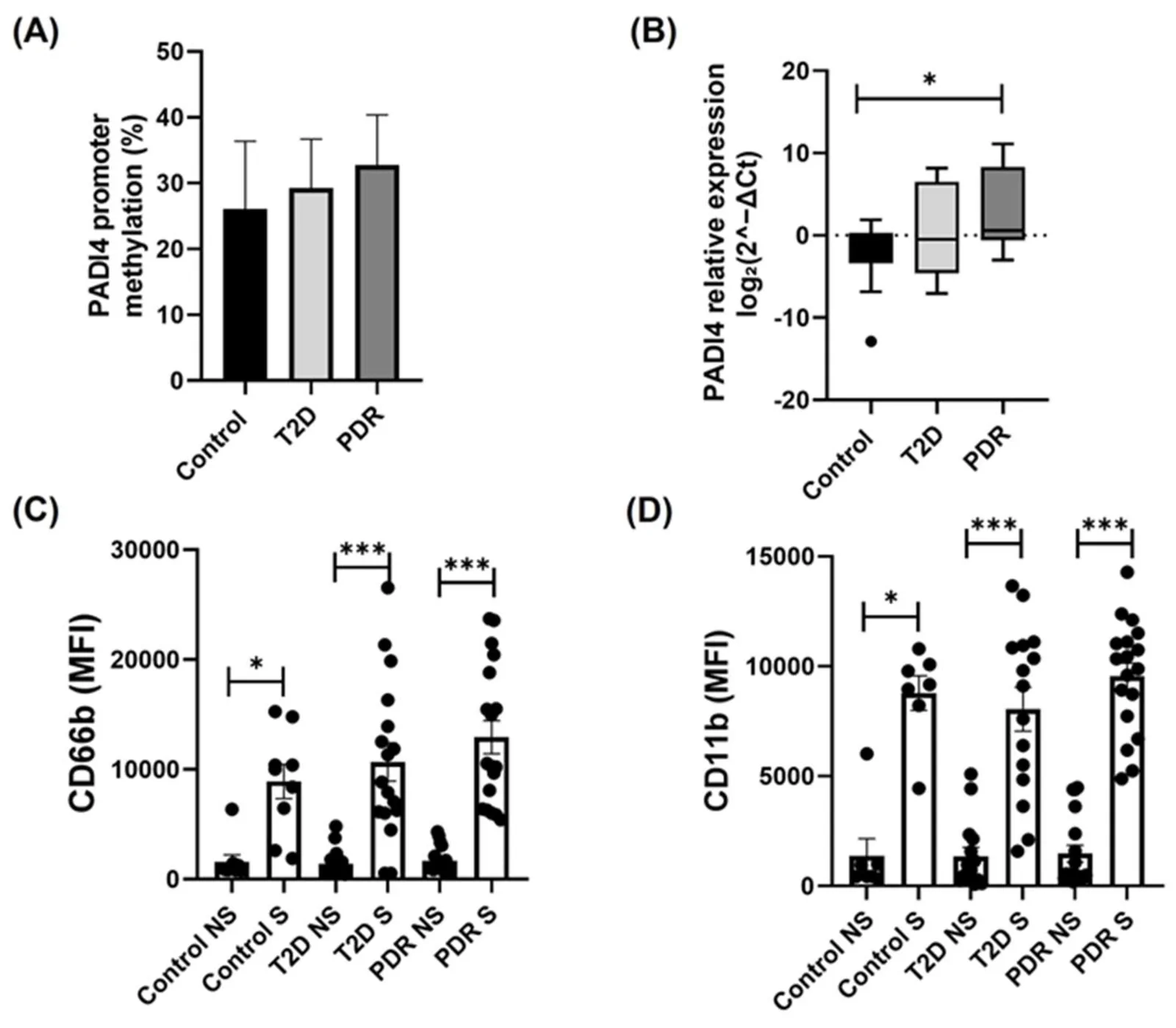
Epigenetic regulation and functional activation of neutrophils in control, T2D, and PDR patients. Percentages of DNA methylation at the *PADI4* promoter region (Met%) in peripheral blood neutrophils from control, T2D, and PDR patients. Methylation was assessed by bisulfite conversion and quantified as the mean percentage of methylated CpG sites. Although slight variations were observed among the groups, no statistically significant differences were detected, indicating that *PADI4* promoter methylation is stable across conditions (**A**). For each sample, ΔCt was calculated as CtPADI4 − CtB2M. Normalized PADI4 expression is presented as −ΔCt, which is mathematically equivalent to log_2_(2^−ΔCt^), such that higher values represent greater PADI4 expression relative to B2M. Box-and-whisker plots showing expression in the control, T2D, and PDR groups. A significant increase in *PADI4* expression was observed in the PDR group compared with the control group (*p* < 0.05), suggesting transcriptional upregulation independent of promoter methylation (**B**). Surface expression of the neutrophil activation markers CD66b (**C**) and CD11b (**D**), as measured by flow cytometry, in peripheral blood neutrophils under nonstimulated (NS) and stimulated (S) conditions. Individual MFI values for all analyzed samples are now shown as separate data points in the corresponding graphs, and the bars represent the mean ± SEM. Stimulation significantly increased the expression of both markers in the control, T2D, and PDR groups, indicating preserved neutrophil activation capacity. No significant differences were observed among the study groups under either condition (**C**,**D**). * *p*  <  0.05; *** *p*  <  0.001.

**Figure 4:**
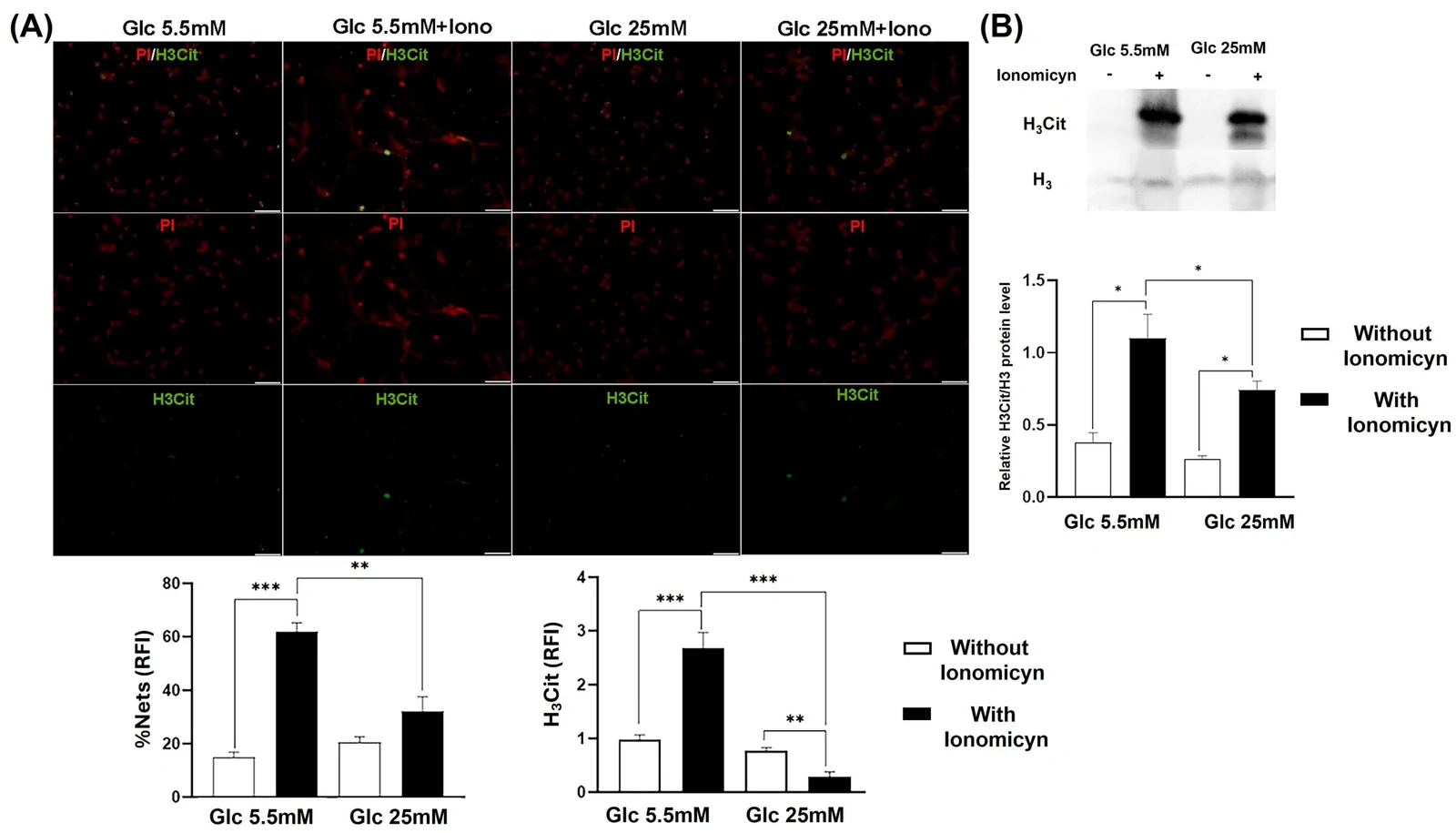
NET formation and histone H3 citrullination are reduced under persistent high-glucose conditions. Peripheral blood neutrophils from healthy donors were incubated for 24 h in normoglycemic (Glc 5.5 mM) or high-glucose (Glc 25 mM) medium and subsequently stimulated with ionomycin (+) or vehicle (–) for 1 h. Representative immunofluorescence images (top) show PI (red) and citrullinated histone H3 (H3Cit; green) staining, indicating NETs and histone citrullination. Quantification (bottom): Left, the percentage of NETs (PI-positive, RFI) significantly increased after ionomycin treatment, with a stronger effect at 5.5 mM glucose. Right, the H3Cit signal (RFI) was markedly induced by ionomycin in normoglycemia but attenuated under high glucose conditions (**A**). The Western blot lanes in panel (**B**) are arranged from left to right as follows: Glc 5.5 mM/ionomycin −, Glc 5.5 mM/ionomycin +, Glc 25 mM/ionomycin −, and Glc 25 mM/ionomycin +. Panel B corresponds to the 24-h glucose-exposure experiment. Western blot analysis of H3Cit and total H3 (loading control) confirmed ionomycin-induced citrullination, with greater levels at 5.5 mM glucose (**B**). The bar graph shows the densitometric quantification of H3Cit normalized to H3. Scale bars = 20 μm. Data are presented as the mean ± SEM. * *p* < 0.05; ** *p* < 0.01; *** *p* < 0.001. *n* = 3.

**Figure 5:**
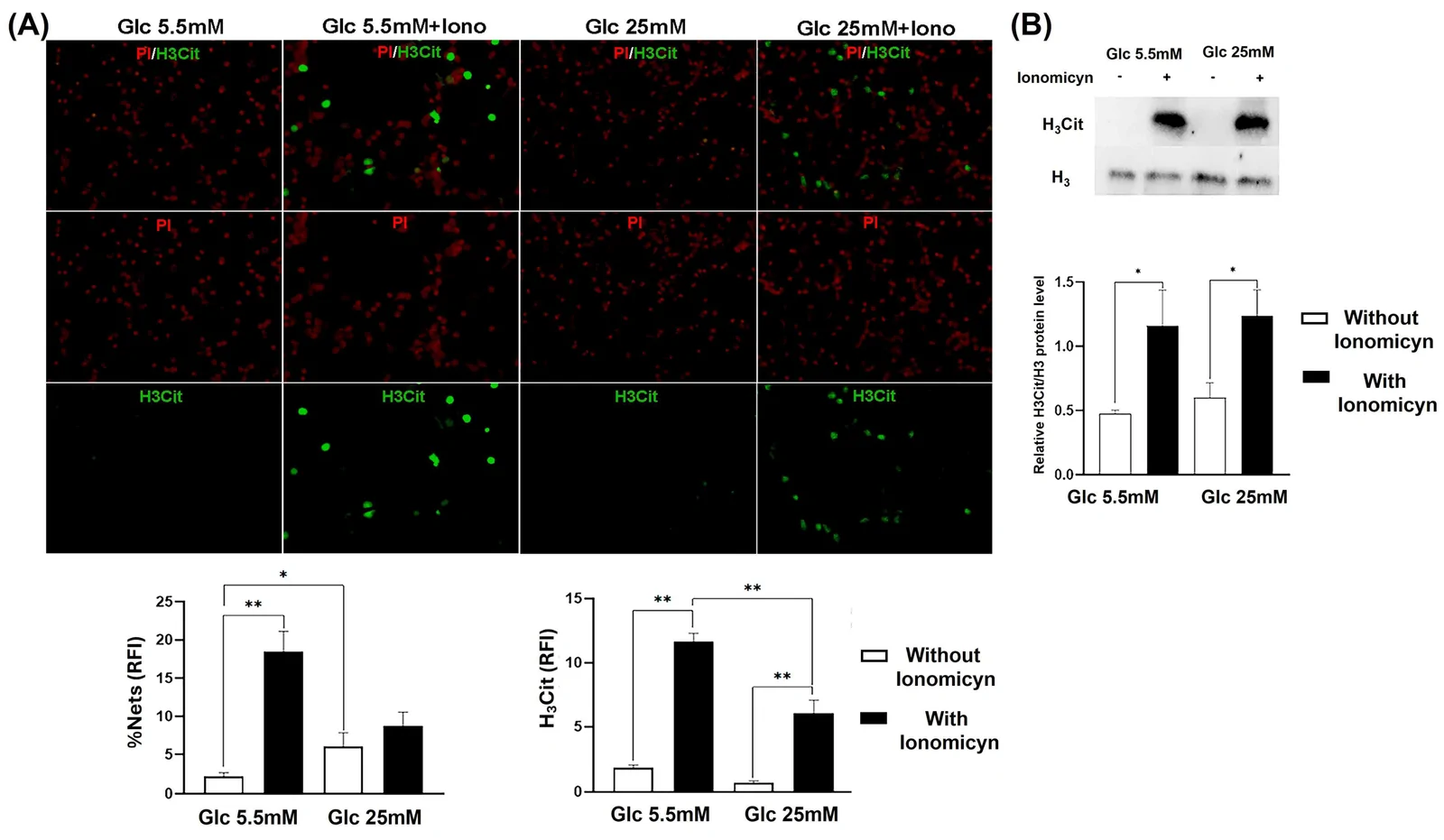
Transient high glucose levels impair NET formation but not histone citrullination in neutrophils. Representative immunofluorescence images of neutrophils cultured under low-glucose (Glc 5.5 mM) or high-glucose (Glc 25 mM) conditions for 1 h in the absence or presence of ionomycin. Cells were stained with propidium iodide (PI; red) and citrullinated histone H3 (H3Cit; green). Quantification of NET formation (left graph) and H3Cit fluorescence intensity (middle graph) revealed that ionomycin markedly increased both parameters under normoglycemic conditions, whereas the effect was attenuated under high glucose conditions (**A**). The Western blot lanes in panel (**B**) are arranged from left to right as follows: Glc 5.5 mM/ionomycin −, Glc 5.5 mM/ionomycin +, Glc 25 mM/ionomycin −, and Glc 25 mM/ionomycin +. Panel (**B**) corresponds to the 1-h glucose-exposure experiment. Western blot analysis of H3Cit and total histone H3 (loading control) (right, upper panel) confirmed increased histone citrullination in the presence of ionomycin, with densitometric quantification shown below (right, lower graph). Data are presented as the mean ± SEM; * *p* < 0.05, ** *p* < 0.01. *n* = 3.

**Table 1:** Demographic, clinical and metabolic characteristics of the subjects included in the study.

	Control	T2D	PDR
*n* = 41	*n* = 9	*n* = 17	*n* = 15
Sex (F:M)	5:4	8:9	6:9
Age	39.7 (±9.5)	53.3 (±9.6) *	56.5 (±13.5) *
T2D diagnosis time	--	7.2 (±4.2) ^a^	20.9 (±6.1) ^a^
BMI	25.7 (±4)	30.4 (±4.6)	28 (±5)
SBP (mmHg)	113.3 (±7.1)	119.3 (±12.4)	126 (±14) *
DBP (mmHg)	78.7 (±5.6)	76.8 (±18.3)	81.8 (±9.8)
Fasting glucose (mg/dL)	86.7 (±4.1)	167.3 (±61.2) *	147.1 (±55.1) *
HbA1c (%)	5.4 (±0.2)	7.9 (±1.9) *	8.7 (±1.9) *
Urea (mg/dL)	25.1 (±6.5)	30.8 (±7.8) *^a^	41.2 (±13.7) ^a^*
Creatinine (mg/dL)	0.75 (±0.1)	0.77 (±0.2)	1.0 (±0.3) *
eGFR (ml/min/1.73 m^2^)	112.8 (±12.4)	103.5 (±20.1) ^a^	80.5 (±26.6) *^a^
BUN	11.7 (±3)	14.4 (±3.6) *^a^	19.5 (±6.4) *^a^
Atherogenic index	3.5 (±0.7)	4.4 (±1.3)	4.6 (±0.9) *
Cholesterol (mg/dL)	176.4 (±18.6)	181.5 (±46.2)	178.4 (±37.3)
HDL-C (mg/dL)	52.5 (±10.8)	47.6 (±28.9) *	42 (±11.9) *
LDL-C (mg/dL)	118.8 (±19)	121.5 (±43.4)	110.9 (±40.1)
Total bilirubins (mg/dL)	0.51 (±0.22)	0.58 (±0.22)	0.36 (±0.18) *
TyG	8.6 (±0.3)	9.5 (±0.7) *	9.5 (±0.6) *
Triglycerides (mg/dL)	120.6 (±35)	185.6 (±79.4)	235.2 (±228.6) *
Uric acid (mg/dL)	5.1 (±1.4)	5.3 (±1.4)	6.2 (±1.8) *
Serum osmolality	296.5 (±2.8)	300.4 (±3.9)	302.9 (±4.3) *

* Statistical difference in comparison with control subjects; ^a^ significant difference between T2D and PDR individuals. BMI, body mass index; SBP, systolic blood pressure; DBP, diastolic blood pressure; HBA1c, glycated hemoglobin; eGFR, estimated glomerular filtration rate; BUN, blood urea nitrogen; HDL-C, high-density lipoprotein cholesterol; LDL-C, low-density lipoprotein cholesterol; TyG, fasting triglyceride to fasting glucose index.

**Table 2:** Associations between NET release and clinical and biochemical parameters in the study cohort.

	NET	
	r	*p*
Age	−0.5386	0.0001
T2D diagnosis time	−0.7074	<0.0001
BMI	−0.1728	0.1399
SBP	−0.3269	0.0185
DBP	−0.2160	0.0874
Fasting glucose	−0.2917	0.0321
HbA1c (%)	−0.4393	0.0020
Urea	−0.5590	0.0001
Creatinine	−0.2746	0.0411
eGFR	0.5051	0.0004
BUN	−0.5590	<0.0001
Atherogenic index	−0.2076	0.0964
Cholesterol	−0.0677	0.3369
HDL-C	0.3525	0.0119
LDL-C	−0.0207	0.4488
Total bilirubin	0.1522	0.1710
Triglycerides	−0.1075	0.2518
TyG	−0.3500	0.0124
Uric acid	−0.4126	0.0037
Serum osmolality	−0.4089	0.0040

BMI, body mass index; SBP, systolic blood pressure; DBP, diastolic blood pressure; HBA1c, glycated hemoglobin; eGFR, estimated glomerular filtration rate; BUN, blood urea nitrogen; HDL-C, high-density lipoprotein cholesterol; LDL-C, low-density lipoprotein cholesterol; TyG, fasting triglyceride to fasting glucose index.

## Data Availability

Data are available upon reasonable request.

## Supplementary Materials

The following supporting information can be downloaded at: https://www.mdpi.com/article/10.3390/biomedicines14102148/s1, Figure S1: Representative flow cytometry histograms of neutrophil activation markers.

## Abbreviations

DR	Diabetic retinopathy
PDR	Proliferative diabetic retinopathy
T2D	Type 2 diabetes
NETs	Neutrophil Extracellular Traps
H3Cit	Histone H3 citrullination
PAD4	Peptidylarginine deiminase 4

## Appendix A

### Appendix A.1. Sequence Primers for Methylation Assays

PADI4

Fwd 5′-AGTTTAGGGGTTTTTTATAGTTAGAGGGAC-3′

Rev 5′-ATCAAAATACCCAACACACACACG-3′

ACTB (CpG-free normalization control)

Fwd 5′-GGTGGTGATGGAGGAGGTTTAG-3′

Rev 5′-CCCTTAAAAATTACAAAAACCACAACC-3′

**Table A1:** Cycling parameter methylation assays.

Temperature (°C)	Time	Cycles
95	3 min	
95	15 s	40
63	60 s	
72	2 min	

HRM analysis was conducted by ramping from 68–80 °C at 0.1 °C/s with continuous fluorescence acquisition.

### Appendix A.2. Sequence of Primers Used for RT-qPCR

PADI4

Fwd 5′-CCCCCAAGGACTTCTTCACAA-3′

Rev 5′-ACCCAAGACTACGCTGCACT-3′

B2M

Fwd 5′-CACCCCCACTGAAAAAGATG-3′

Rev 5′-ATATTAAAAAGCAAGCAAGCA-3′

**Table A2:** Cycling conditions for RT-qPCR assays.

Temperature (°C)	Time	Cycles
42	5 min	
95	3 min	
95	5 s	35
60	20 s	
72	20 s	
