# Supplementary material for: Stimulus-Induced Histone H3 Citrullination and Neutrophil Extracellular Trap Formation Are Impaired in Patients with Proliferative Diabetic Retinopathy
Source: Biomedicines. 2026 Sep 23;14(10):2148. doi: 10.3390/biomedicines14102148 (PMC13701284; doi:10.3390/biomedicines14102148)
Supplement: Supplementary file 1 [file biomedicines-14-02148-s001.zip › biomedicines-4471549-supplementary.pdf]

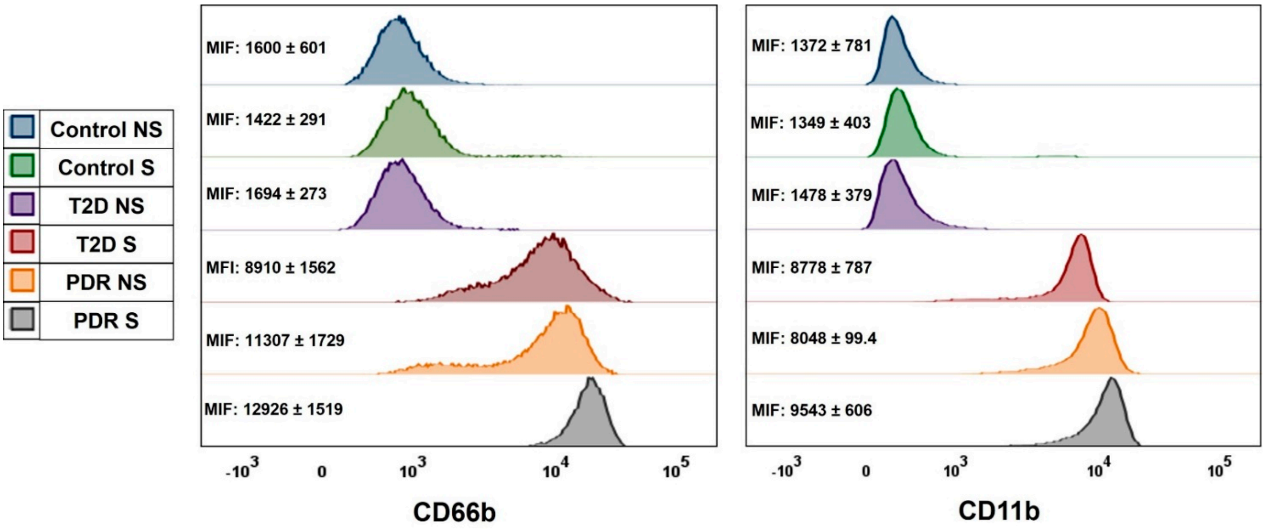

Supplementary Figure S1. Representative flow cytometry histograms of neutrophil activation markers. *Representative fluorescence-intensity histograms show the surface expression of CD66b (left) and CD11b (right) in neutrophils from the Control, type 2 diabetes mellitus (T2D), and proliferative diabetic retinopathy (PDR) groups under non-stimulated (NS) and stimulated (S) conditions. Histograms are displayed vertically for clarity, and fluorescence intensity is presented on a logarithmic scale. The corresponding group mean fluorescence intensity (MFI) values are embedded within each histogram and expressed as mean ± SEM. Colors identify the experimental groups and conditions as follows: Control NS, blue; Control S, green; T2D NS, purple; T2D S, red; PDR NS, orange; and PDR S, gray. Stimulation produced a rightward shift in the fluorescence distributions of both CD66b and CD11b in all three groups, consistent with increased surface expression of these neutrophil activation markers. The complete quantitative analysis and individual MFI values are presented in Figure 3C and 3D.*
